# Transcription Factor–Based Classification of Pituitary Neuroendocrine Tumors: Practical Immunohistochemical Algorithms, Molecular Correlates, and Diagnostic Challenges in the 5th WHO Era

**DOI:** 10.3390/ijms27052307

**Published:** 2026-02-28

**Authors:** Nirmal Pandit, Yahya Wehbeh, Omar Itani, Dimitrios Kanakis

**Affiliations:** 1 Laboratory of Pathology, Department of Basic and Clinical Sciences, University of Nicosia Medical School, Nicosia 2408, Cyprus; pandit.n@live.unic.ac.cy (N.P.); wehbe.y@live.unic.ac.cy (Y.W.); itani.o1@live.unic.ac.cy (O.I.); 2 Centre of Neuroscience and Integrative Brain Research (CENIBRE), University of Nicosia Medical School, Nicosia 2408, Cyprus

**Keywords:** pituitary neuroendocrine tumors, transcription factor-based classification, immunohistochemistry, PitNETs, PIT1, TPIT, WHO 5th edition classification

## Abstract

Pituitary neuroendocrine tumors (PitNETs) constitute a significant proportion of primary intracranial neoplasms and were historically differentiated based on clinical hormone excess syndromes and tinctorial properties. The 5th edition of the WHO classification introduces a paradigm shift towards the lineage-based taxonomy based on the cell-specific expression of transcription factors (TFs). This overview focuses on the biological justifications and diagnostic value of the core TFs of Pituitary-Specific Positive Transcription Factor 1 (PIT1), T-Box Pituitary Transcription Factor (TPIT), and Steroidogenic Factor 1 (SF1), which signify the somatotroph, lactotroph, thyrotroph, corticotroph, and gonadotroph lineages, respectively. By focusing on TF expressions instead of hormone immunoreactivity, pathologists can better subtype clinically non-functioning tumors, effectively relegating the previously overutilized null cell category to about 1% of cases. The TF-based classification is also essential in discriminating high-risk histotypes of silent corticotroph tumors, sparsely granulated somatotrophs, and immature PIT1-lineage PitNETs, which are linked to a higher invasiveness and recurrence. We suggest a practical, stepwise immunohistochemical diagnostic algorithm with the integration of ancillary markers (e.g., GATA3 and ERα) to refine lineage assignment. New molecular correlates such as *GNAS* and *USP8* mutations also add to this framework and guide the use of individualized treatment involving somatostatin analogs or dopamine agonists. And lastly, we discuss the ongoing issues of diagnosis of triple-negative and multilineage tumors and the growing importance of DNA methylation profiling and artificial intelligence in standardized reporting and improving precision management.

## 1. Introduction

Pituitary neuroendocrine tumors (PitNETs) are epithelial neuroendocrine neoplasms (NENs) that arise from adenohypophyseal cells [1]. Pituitary tumors make up about 10–15 percent of all intracranial neoplasms, making them the second most common primary intracranial neoplasm and the most common neuroendocrine neoplasm in the human body [2,3]. It is estimated that clinically relevant PitNETs occur at a prevalence of 78–116 cases per 100,000 people [1,2,3]. Patients typically present either with sellar mass effects, such as visual impairment, headache, or hypopituitarism, or with syndromes due to hormonal hypersecretion, such as acromegaly or Cushing’s disease [3,4,5]. Although the majority of PitNETs are non-cancerous and clinically indolent, there are subsets that demonstrate significant variability and aggressiveness with invasive growth, high proliferation, and resistance to standard treatments [5,6,7].

In the past, adenohypophyseal neoplasms were referred to as pituitary adenomas (PAs) [6]. Initial taxonomies were simple, as they were mostly based on the tinctorial properties of acidophils, basophils, and chromophobes [4,8]. Further developments in pathology led to the shift towards a morpho-functional paradigm, which combined clinical status (functional or non-functional), hormonal profiles (immunohistochemistry), and ultrastructure (electron microscopy) [4,8,9]. On this basis, the 2004 World Health Organization (WHO) classification defined seven main types—prolactin (PRL), growth hormone (GH), adrenocorticotropic hormone (ACTH), thyroid-stimulating hormone (TSH), gonadotropin (LH/FSH) secreting tumors, null cell, and plurihormonal tumors—and subdivided them into 13 ultrastructural types [4]. Despite this, the hormonal classification had significant limitations. The common use of the term ‘pituitary adenoma’ was problematic because it implied a purely benign behavior, which contradicted the existence of tumors that are locally invasive, clinically aggressive, or, in rare cases, metastatic [4,6,7,8]. The WHO 2004 classification introduced the category ‘atypical adenoma’, defined by proliferation markers such as Ki-67 index of >3% or diffuse p53 staining. However, this category has proven unreliable due to inconsistent prognosis [4,5,6,10]. It also included the poorly defined category of null cell adenoma (non-secretory adenoma) [4,9]. The weaknesses of hormone immunohistochemistry (IHC) suggested that the use of secretory products alone cannot be enough to capture the complexity of tumor cell lineage.

As a response to these limitations, in 2017, the International Pituitary Pathology Club suggested the new term “Pituitary Neuroendocrine Tumor (PitNET)” as more reflective of the epithelial neuroendocrine etiology of such tumors and their spectrum of behavior, ranging from indolent to locally invasive [1,3,4,5,8,11,12]. This nomenclature has since been preferred in the 5th edition of the WHO Classification of Endocrine and Neuroendocrine Tumors (2022), although the term “pituitary adenoma” is acceptable [3,5,11]. This modern classification identifies the cell lineage of a tumor by the transcription factor (TF) IHC. Three major TF families are currently in common use in diagnostic IHC: PIT1 for somatotroph, lactotroph, or thyrotroph lineage, TPIT for corticotroph lineage, and SF-1 (Steroidogenic Factor 1) for gonadotroph lineage, along with supporting markers such as GATA3 (GATA Binding Protein 3) and ERα (Estrogen Receptor Alpha) [4,10,11,12]. The implementation of TF-based IHC has enabled more accurate subtyping of PitNETs. It allows reclassification of many tumors previously labeled as ‘null-cell tumors’ as gonadotroph PitNETs and helps define high-risk subtypes such as sparsely granulated somatotroph, silent corticotroph, Crooke cell, and immature PIT1-lineage tumors, with important implications for prognosis and therapy [2,4,5,9,11,12,13].

However, there are still practical issues, including limitations in IHC standardization (e.g., fixation protocols, cut-off levels) and the unreliability of antibody supply (e.g., SF-1 and TPIT). TF-negative tumors (null-cell tumors) and rare multilineage tumors (e.g., PIT1 -SF1 co-expressions) still pose a challenge to the existing classification system, so they require further molecular research [4,9,11,12,13].

This review provides a recent development in the practical overview of TF-based classification of PitNETs in the 5th WHO era, focusing on the biology of lineage-defining transcription factors, stepwise immunohistochemical algorithms, molecular and clinicopathologic correlates, and common diagnostic challenges. Emphasis is placed on pragmatic guidance for routine surgical pathology and multidisciplinary decision-making.

Several recent reviews have summarized the conceptual aspects of the WHO 2022 TF-based PitNET classification, but have provided less practical guidance for day-to-day diagnostic work-up and reporting [3,5,6,10]. In contrast, the present review aims to integrate the biology of lineage-defining TFs with a stepwise immunohistochemical algorithm, a minimum reporting dataset, and explicit links between TF-defined histotypes, molecular drivers, and clinical behavior. In particular, we emphasize the recognition and management implications of lineage-associated histotypes with unfavorable clinical behavior (e.g., sparsely granulated somatotroph, silent corticotroph, Crooke cell, and immature PIT1-lineage tumors) and summarize key differences in their classification and clinical impact in a comparative table.

## 2. Historical and Conceptual Background

This section reviews the evolution of pituitary tumor classification from early tinctorial and hormone-based schemes to the current WHO 5th edition framework, highlighting the limitations that motivated a lineage-based, transcription factor–centered approach.

### 2.1. Evolution of Pituitary Tumor Classification

The classification of adenohypophyseal neoplasms has undergone a significant change, evolving from systems based largely on clinical and morphological criteria to frameworks incorporating immunohistochemical and molecular stratification [5,7,9]. Initial classification systems were largely established based on the clinical presentation of one of the hormone excess syndromes, such as acromegaly, hyperthyroidism, or Cushing’s disease [5]. Early classification schemes also relied on tinctorial properties of the neoplastic cells, using conventional stains such as hematoxylin–eosin to separate tumors into acidophilic, basophilic, or chromophobic types. This early classification, however, was subjective and poorly reproducible [14].

The classification system progressed with the establishment of IHC, which provided the possibility of directly detecting the pituitary hormones in the tumor cells (e.g., GH, PRL, ACTH, TSH, FSH, LH) [3,10,14]. IHC enabled a more precise matching of tumor histology with their clinical endocrine syndromes and made it possible to classify tumors by simply the hormone that they synthesize (e.g., a GH-producing adenoma) [3,10].

Despite these developments, the hormone-based classification had some strong clinicopathological contradictions [1]. Many clinically non-functioning PitNETs (NF-PitNETs), without overt clinical manifestations of hormonal hypersecretion, were found to be positive for at least one pituitary hormone on IHC. Such tumors were referred to as silent neoplasms or silent adenomas [15]. An example of such a tumor is a silent corticotroph PitNET (historically ‘silent corticotroph adenoma’), which produces ACTH but does not induce hypercortisolism [12,15]. Besides, the tumors that contained no identifiable anterior pituitary hormones using IHC were classified into the ambiguous group of null cell adenomas [14,15]. These gaps in the earlier classification highlight the necessity of a paradigm of classification based on the fundamental biology of pituitary cell differentiation and not necessarily based on hormone detection only. The major changes in the WHO classification of pituitary tumors over time are summarized in Table 1.

### 2.2. Rationale for Lineage-Based, TF-Centered Classification

The limitations of hormone-based classification systems prompted a shift toward cell lineage–based classification using pituitary-specific transcription factors (TFs). TFs are regulatory proteins important for embryogenesis and differentiation of adenohypophyseal cells and regulate the expression of the hormone-encoding genes. This classification system recognizes three major cell lineages, each of which is controlled by a specific core TF [10,12,15]:

1. PIT1-lineage specifies somatotrophs (GH-secreting), lactotrophs (PRL-secreting), and thyrotrophs (TSH-secreting).
2. TPIT-lineage specifies corticotrophs (ACTH-secreting).
3. SF1-lineage specifies gonadotrophs (FSH/LH-secreting).

This lineage-based approach was adopted in the 2017 WHO classification of pituitary tumors and further refined in the 2022 WHO Classification of Endocrine and Neuroendocrine Tumors. In the 2017 WHO classification, the term ‘pituitary neuroendocrine tumor’ (PitNET) was introduced to align pituitary tumors with the broader classification of neuroendocrine neoplasms [1,3,5,15]. The incorporation of TFs has revolutionized the diagnosis of PitNETs, especially the non-functioning tumors. The systematic use of TF IHC has shown that a large proportion of tumors previously diagnosed as null cell adenomas actually represent silent lineage-specific tumors [1,9,12]. This observation explains why TF-based classification can be used to represent cellular origin more accurately than hormone expression only [3].

TF-based classification is important for the recognition and classification of the high-risk subtypes regardless of clinical hormonal activity. The WHO classification associates several histological subtypes with potentially aggressive behavior, and a TF-based classification is essential for their identification [5]:

1. PIT1-lineage aggressive tumors include sparsely granulated somatotroph tumors (more aggressive and often resistant to somatostatin analogue (SSA) therapy than their densely granulated counterparts), acidophil stem cell tumors, and immature PIT1-lineage tumors (formerly classified as silent subtype 3 adenomas with poor differentiation and increased potential for recurrence and invasiveness).
2. TPIT-lineage aggressive tumors include silent corticotroph tumors (that are more likely to exhibit enhanced proliferative and invasive capacity compared to silent gonadotroph tumors), and the rare but highly aggressive Crooke cell tumors.

TF-based classification provides a basis for predicting tumor behavior, responsiveness to medical treatment, and overall prognosis by assigning each PitNET to a specific cell lineage (PIT1, TPIT, or SF-1) [1].

## 3. Biology of Pituitary Lineage-Defining Transcription Factors

Anterior pituitary cells arise through a tightly regulated developmental cascade that begins in Rathke’s pouch progenitors and proceeds through a series of increasingly restricted states. Early patterning factors such as HESX1 and the LIM-homeodomain proteins LHX3 and LHX4 establish a pituitary primordium, within which multipotent progenitors progressively commit to distinct endocrine lineages [12,17]. At this stage, the balance between proliferation and differentiation is controlled by a network of transcription factors (TFs) that both maintain progenitor pools and initiate lineage-restricted programs of hormone gene expression [10,12]. As development proceeds, key “gatekeeper” TFs—most notably PROP1 (Prophet of PIT1), TBX19 (TPIT), and NR5A1 (SF-1)—play a hierarchical role in directing progenitors toward PIT1-, TPIT-, or SF-1-defined lineages, respectively, while suppressing alternative fates [12,17].

The lineage-defining TFs PIT1 (POU1F1), TPIT (TBX19), and SF-1 (NR5A1) are then maintained in differentiated somatotroph, lactotroph, thyrotroph, corticotroph, and gonadotroph cells, where they regulate hormone-encoding genes and many aspects of cell identity and function [10,12,15,17]. In PitNETs, these TFs are generally preserved even when hormone production is reduced, inefficient, or clinically silent, making them more reliable indicators of lineage than hormone immunostaining alone [1,3,5]. The 5th edition of the WHO Classification, therefore, places these lineage-defining TFs at the center of diagnostic algorithms, using them as robust surrogates for the underlying developmental program from which each PitNET arises [1,3,5]. The following subsections summarize the biology and clinicopathologic correlates of each lineage, focusing on how PIT1-, TPIT-, and SF-1–driven programs shape tumor phenotype and behavior.

### 3.1. PIT1 (POU1F1) and PIT1-Lineage Tumors

Within this developmental framework, PIT1 (also known as POU1F1, POU class 1 homeobox 1) is induced downstream of PROP1 in committed PIT1-lineage precursors and is then maintained in differentiated somatotroph, lactotroph, and thyrotroph cells [12,17]. PIT1 is a nuclear transcription factor of the POU-domain family that is necessary for the differentiation, proliferation, and survival of these anterior pituitary cell types [12,17]. During pituitary embryogenesis and in the adult gland, PIT1 regulates the expression of GH, PRL, and TSH, thereby coupling lineage identity to hormone production [12].

PIT1-lineage PitNETs are divided into subgroups based on granulation pattern, hormone expression, and cytoskeletal features [5,14]. Somatotroph PitNETs are classified into densely granulated and sparsely granulated tumors. Densely granulated tumors are composed of GH-positive acidophilic cells with a characteristic perinuclear keratin pattern, whereas sparsely granulated tumors often have chromophobic cytology and contain fibrous bodies (cytoplasmic keratin aggregates) and are associated with more aggressive behavior and a poorer clinical response to first-generation somatostatin analogs. Lactotroph PitNETs are mostly sparsely granulated and show a typical juxtanuclear “dot-like” immunostaining pattern for PRL, while the rare densely granulated forms demonstrate diffuse cytoplasmic PRL expression. Thyrotroph PitNETs are rare and often display fibrotic or spindle-cell morphology; they secrete TSH and are positive for both PIT1 and GATA3 on immunohistochemistry. Among plurihormonal PIT1-lineage tumors, immature PIT1-lineage tumors and acidophil stem cell tumors are often large and clinically aggressive, whereas mature plurihormonal PIT1-lineage tumors show more variable behavior. These entities exhibit lineage infidelity, with co-expression of several PIT1-lineage hormones without full terminal differentiation.

**Figure 1:**
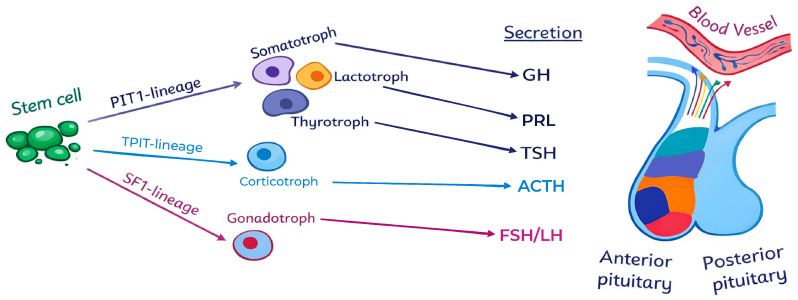
Anterior pituitary cell lineages and their hormones. This schematic provides a simplified overview of the differentiation of anterior pituitary cell lineages (PIT1, TPIT, and SF-1) and their associated hormones and does not depict the full complexity of pituitary embryogenesis or intermediate progenitor states. The different colors within the anterior pituitary represent different cell populations corresponding to the left side of the image.

The main anterior pituitary cell lineages and their associated hormones are illustrated in Figure 1.

### 3.2. TPIT (TBX19) and Corticotroph Tumors

T-box pituitary transcription factor (TPIT), encoded by the T-Box Transcription Factor 19 (TBX19) gene, is the master regulator of the corticotroph lineage and controls the final differentiation of cells that produce pro-opiomelanocortin (POMC) [18,19]. TPIT binds to the POMC promoter together with other transcription factors, including Pitx1 (Pituitary Homeobox 1), to drive the production of ACTH [12,18].

TPIT-lineage PitNETs are typically divided into two clinicopathological categories:

ACTH-secreting PitNETs (Densely granulated) are the major etiological agents of Cushing’s disease and present as small, basophilic microadenomas with strong periodic acid-Schiff (PAS) staining [5,14,19].

Sparsely granulated or silent corticotroph tumors (SCTs) are clinically non-functional but immunoreactive to TPIT and ACTH [5,20]. These tumors present as macroadenomas at the time of diagnosis and portray more aggressive clinical behaviors, such as greater recurrence and invasion rates as compared to gonadotroph tumors [19].

Within this lineage, a distinctive variant termed Crooke cell PitNET is recognized. It is a highly aggressive corticotroph tumor characterized by extensive perinuclear hyaline (keratin) ring-like cytoplasmic change in the majority of tumor cells. The hyaline changes indicate a paradoxical state of cells, where the cells exhibit characteristics of the hormone-mediated feedback inhibition and at the same time show elevated proliferative indices and invasive potential [5].

Melanotrophs also belong to the TPIT/POMC lineage and give rise to a rare tumor that produces alpha-melanocyte-stimulating hormone (a-MSH). This tumor can be distinguished from corticotroph tumors in terms of its location (Pars intermedia) and negative ACTH immunoreactivity [18].

### 3.3. SF1 (NR5A1) and Gonadotroph Tumors

SF1, encoded by the NR5A1 (Nuclear Receptor Subfamily 5 Group A Member 1) gene, is a nuclear receptor TF that plays a crucial role in the differentiation and functional regulation of the gonadotroph lineage, which produces FSH and LH [12,21]. Unlike the PIT1 and TPIT lineages, which are often characterized by clinical endocrine syndromes, SF1-lineage PitNETs are, in general, clinically non-functional and are only revealed through the presence of mass effect symptoms [12,22].

SF1-lineage PitNETs constitute the majority of clinically non-functioning pituitary adenomas [5]. These tumors usually show patchy or weakly stained FSH, LH, and the alpha-subunit glycoprotein on IHC. But diffuse nuclear SF1 expression is the most sensitive and specific marker for identifying these tumors. Even though these tumors tend to be quite large, they tend to exhibit a more indolent behavior with reduced recurrence rates after complete resection compared to silent corticotroph or immature PIT1-lineage tumors [14,20].

### 3.4. Supporting and Additional Markers: GATA3, ERα, Others

The diagnostic accuracy of TF-based classification can be further enhanced by ancillary markers that are used for (1) routine support of lineage assignment and (2) more specialized developmental or contextual information.

1. Markers supporting routine lineage assignment:

GATA binding protein 3 (GATA3) is a nuclear protein expressed in a subset of PIT1-lineage tumors, particularly thyrotroph PitNETs, and can also be detected in some gonadotroph tumors [17]. It is a useful adjunct marker for confirming thyrotroph differentiation (co-localizing with PIT1) and can aid in lineage assignment when SF-1 immunostaining is inconclusive [12].

Estrogen receptor alpha (ERα) is a TF that plays a crucial role alongside PIT1 in the differentiation and maintenance of lactotrophs and mammosomatotrophs [17]. Its expression has been associated with higher Dopamine Receptor D2 (DRD2) expression and may contribute to the good clinical responsiveness of many lactotroph tumors to dopamine agonist therapy [20].

2. Developmental and contextual markers used in selected cases

Other TFs, including PROP1, PITX1, and PITX2 (Pituitary Homeobox 2), are not typically required for routine lineage assignment but can provide additional insight in selected or research settings. PROP1 is an early developmental TF that precedes PIT1 and is upregulated in certain immature PitNETs; it is considered a marker of a progenitor-like state rather than a terminally differentiated lineage. PITX1 and PITX2 complement TPIT in corticotroph differentiation, especially by activating POMC gene expression, and help to contextualize corticotroph tumors within normal pituitary developmental pathways [12].

### 3.5. Link with Germline and Somatic Driver Events

Molecular alterations in PitNETs broadly align with TF-defined cell lineages and help explain their endocrine and clinical behavior. In the PIT1 lineage, gain-of-function mutations in the Guanine Nucleotide-binding Protein alpha stimulating (*GNAS*) gene have been detected in up to 40 percent of somatotroph tumors, particularly in densely granulated subtypes, where constitutive activation of Gs-cAMP signaling drives GH hypersecretion [20,22]. *GNAS*-mutated somatotroph PitNETs are typically smaller and show increased responsiveness to somatostatin analog therapy, illustrating how a specific signaling alteration translates into both a characteristic biochemical phenotype and a favorable treatment response [20,22]. By contrast, germline alterations, such as Aryl hydrocarbon receptor Interacting Protein (*AIP*) mutations (often associated with young-onset, large somatotroph PitNETs) and Multiple Endocrine Neoplasia Type 1 (MEN1) mutations (frequently associated with lactotroph and other PitNETs) are enriched in tumors with more aggressive clinical behavior, linking inherited defects in tumor suppressor pathways to earlier presentation, larger tumor burden, and higher recurrence risk within the PIT1-lineage spectrum [5,22,23].

In the TPIT-lineage, gain-of-function somatic *USP8* mutations are detected in a substantial proportion (approximately 30 to 50 percent) of functioning corticotroph tumors [20,22]. These mutations impair epidermal growth factor receptor (EGFR) degradation, prolonging EGFR signaling and thereby stimulating POMC transcription and ACTH hypersecretion, which underlies the biochemical phenotype of Cushing’s disease in many corticotroph PitNETs [19,20,22]. In Ubiquitin-Specific Protease 48 (*USP8*) wild-type tumors, other molecular alterations—including *USP48* and *BRAF* (B-Raf Proto-Oncogene) mutations—also converge on enhanced Mitogen-Activated Protein Kinase (MAPK) pathway activity and POMC promoter activation, providing a mechanistic explanation for ACTH overproduction in a broader subset of TPIT-lineage neoplasms [19,22].

By contrast, SF-1-lineage PitNETs have not been linked to a single dominant recurrent driver event; instead, they form distinct clusters in methylation and transcriptomic analyses, while their somatic mutational landscape remains relatively heterogeneous and less well characterized [22]. These epigenetic and transcriptional signatures support the notion that SF-1-lineage (gonadotroph) PitNETs represent a biologically coherent group within which variable growth dynamics and clinical courses likely reflect a combination of lineage context and diverse, lower-frequency molecular alterations [22].

## 4. Practical TF-Based Immunohistochemical Classification

This section translates the lineage-based framework into a practical immunohistochemical work-up for pituitary region neoplasms, outlining recommended panels, stepwise diagnostic algorithms, and key distinctions between PitNETs and TTF-1–positive posterior pituitary tumors.

### 4.1. Recommended IHC Panels and Technical Issues

In the 5th edition of the WHO Classification, accurate diagnosis of PitNETs requires a shift from hormone-only panels to a lineage-based diagnostic approach using transcription factors (TFs). The minimal recommended panel of any pituitary neoplasms should include the three fundamental TFs (PIT1, TPIT, and SF1) as well as pan-neuroendocrine markers such as synaptophysin and chromogranin A [5,24,25]. Ki-67 can be used to assess proliferative activity, although PitNETs are not graded into G1–G3 categories as in other NENs [5,26]. For a comprehensive evaluation, an extended immunohistochemical panel is recommended that includes GATA3, ERα, and low molecular weight cytokeratin (LMWCK) such as CAM5.2 clone, to the core TF and neuroendocrine markers [5,14,25]. The composition of the minimal and extended panels is summarized in Table 2.

There are significant challenges in technical execution and interpretation. Pre-analytic variables, such as tissue fixation and antigen retrieval, are critical. Prolonged formalin fixation can cause extensive cross-linking and epitope masking, leading to reduced antigen detectability and false-negative staining [10,27]. Pitfalls include patchy or weak nuclear staining that may be observed in gonadotroph tumors with SF1 expression. In addition, a universal cut-off point of TF positivity does not exist; different studies have used various cut-offs ranging from 5 to 80 percent, while some studies have used a proportion system [9].

### 4.2. Stepwise Algorithm for Work-Up of a Pituitary Region Neoplasm

In routine practice, the diagnostic work-up of a pituitary region neoplasm can be approached as a simple, stepwise immunohistochemical workflow:

Step 1—Confirm neuroendocrine nature on H&E and pan-neuroendocrine markers.

The work-up starts with Hematoxylin and Eosin (H&E) stains to verify the presence of a monomorphic population of cells with “salt and pepper” chromatin and an organoid or trabecular growth pattern typical of neuroendocrine differentiation. First-line immunohistochemistry includes synaptophysin and chromogranin A to confirm neuroendocrine lineage and distinguish PitNETs from non-neuroendocrine sellar lesions [25].

Step 2—Apply the core TF and pituitary hormone panel.

Once a pituitary neuroendocrine neoplasm has been established, the core panel of lineage-defining TFs (PIT1, TPIT, and SF1) is used to place the tumor into one of the three major lineages [14,25]. PIT1-positive tumors are further categorized as somatotroph, lactotroph, thyrotroph, or plurihormonal PIT1-lineage tumors, whereas TPIT- and SF1-positive tumors define corticotroph and gonadotroph PitNETs, respectively [14,25]. Pituitary hormone immunohistochemistry then refines this assignment by documenting which of the six anterior pituitary hormones (GH, PRL, ACTH, TSH, FSH, and LH) and the α-subunit are expressed and whether staining is diffuse or focal.

Step 3—Refine lineage assignment with ancillary markers.

Lineage assignment can be further refined using ancillary markers. GATA3 and ERα support PIT1-lineage assignment, particularly in thyrotroph and lactotroph/mammosomatotroph tumors, while low–molecular weight cytokeratin (LMWCK) such as CAM5.2 highlights perinuclear fibrous bodies in sparsely granulated somatotroph PitNETs and other characteristic cytoskeletal patterns [5,12,27]. These ancillary markers improve the recognition of subtypes with important prognostic and therapeutic implications.

Step 4—Consider alternative diagnoses in TF-negative or unusual phenotypes.

**Figure 2:**
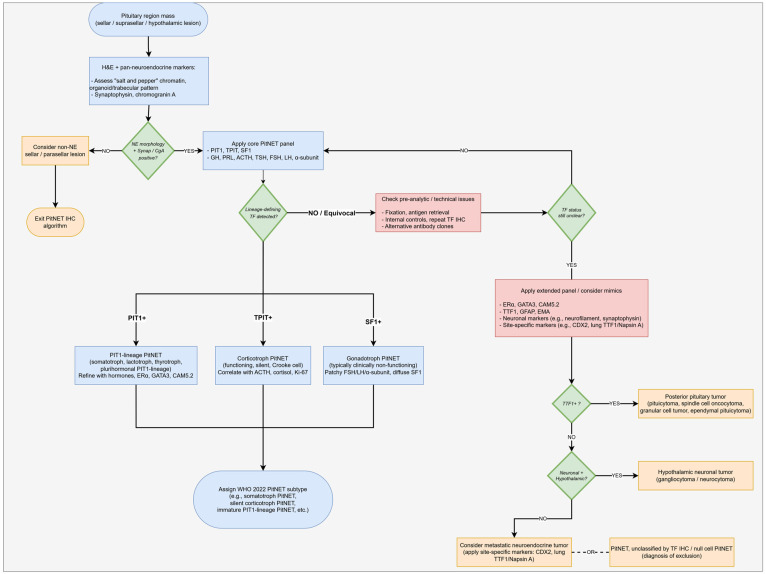
Stepwise immunohistochemical algorithm for the diagnostic work-up of pituitary region neoplasms. The diagnostic workflow begins with confirmation of neuroendocrine differentiation on hematoxylin and eosin (H&E) sections and pan-neuroendocrine markers (synaptophysin, chromogranin A), followed by application of the core lineage-defining transcription factors (PIT1, TPIT, and SF1) together with the six anterior pituitary hormones (GH, PRL, ACTH, TSH, FSH, and LH) and the α-subunit to assign tumors to PIT1-, TPIT-, or SF1-lineages. Ancillary markers such as ERα, GATA3, and low-molecular-weight cytokeratin (CAM5.2) are then used to refine subtype assignment, particularly for sparsely granulated somatotrophs and other PIT1-lineage PitNETs, and to highlight patterns with prognostic and therapeutic relevance. In cases that remain TF-negative or immunophenotypically ambiguous after repeat immunohistochemistry, an extended panel (including TTF1, GFAP, EMA, neuronal markers, and site-specific markers such as CDX2 or lung TTF1/Napsin A) and clinicoradiologic correlation guide reclassification toward posterior pituitary tumors, hypothalamic neuronal tumors, metastatic neuroendocrine neoplasms, or, rarely, a PitNET unclassified by TF immunohistochemistry/null cell PitNET, which should be regarded as a diagnosis of exclusion. Arrows indicate the direction of diagnostic workflow; rectangles represent process steps and diamonds decision points; blue boxes denote the core PitNET workflow, pink boxes technical/troubleshooting steps, and orange boxes alternative or non-PitNET diagnostic endpoints.

If the tumor appears negative for all core TFs, pathologists should first exclude posterior pituitary tumors (PPTs) and other non-adenohypophyseal sellar lesions; a diagnosis of true null cell PitNET should be reserved for rare cases in which no lineage-defining TF or alternative origin can be demonstrated [5,9]. In this setting, additional markers such as TTF1 (for pituicyte-derived posterior pituitary tumors), GFAP (for glial lesions), and neuronal markers such as neurofilament (for gangliocytomas or neurocytomas) are particularly informative [5,14,28]. A schematic representation of this stepwise immunohistochemical workflow is provided in Figure 2.

Common diagnostic pitfalls arising from genuinely triple-negative tumors, multilineage or mixed-TF PitNETs, and discordant hormone/TF profiles are discussed in detail in Section 5.1, Section 5.2 and Section 5.3, as they often require repeat staining, an extended panel, or even methylation profiling to achieve accurate classification.

### 4.3. Handling Discordant or Non-Diagnostic TF Results

In routine practice, TF immunostains are not infrequently weak, patchy, or discordant with hormone expression, and a structured approach is required before concluding that a PitNET is truly TF-negative or unclassifiable. A practical stepwise strategy is as follows:

First, technical issues should be excluded. Internal positive controls (residual non-neoplastic adenohypophyseal cells) and external control tissues must be carefully evaluated to confirm adequate fixation, antigen retrieval, and antibody performance [9,10]. In the presence of globally weak or absent nuclear staining in both tumor and control tissue, repeat staining with optimized protocols or alternative antibody clones is preferable before interpreting the result as biologically negative [9,10,25].

Second, the morphology and core immunophenotype should be re-examined in parallel. The architectural pattern, cytology, and pan-neuroendocrine markers (synaptophysin, chromogranin A) should be integrated with hormone IHC, looking for focal lineage-specific hormone expression that may guide interpretation of weak TF staining [5,14,25]. Special attention should be paid to the possibility of entrapped normal adenohypophyseal acini, which can show strong hormone and TF expression and must not be overinterpreted as part of the tumor [5].

Third, an extended panel should be deployed when TF results remain equivocal. Auxiliary markers, such as ERα and GATA3 can support PIT1-lineage assignment, while LMWCK (e.g., CAM5.2) highlights fibrous bodies in sparsely granulated somatotroph PitNETs and other characteristic cytoskeletal features [5,12,27]. Markers including TTF-1, GFAP, EMA, and neuronal markers (e.g., neurofilament) help redirect the diagnosis toward posterior pituitary tumors, meningioma, or hypothalamic neuronal tumors when appropriate [5,14,28]. If, after adequate technical validation and expanded IHC, TF expression remains absent or non-diagnostic, a cautious designation, such as “PitNET, unclassified by TF IHC” or “possible null cell PitNET (diagnosis of exclusion)” is preferable to inferring a specific lineage [5,9]. In particularly challenging cases, referral to a reference center or the use of DNA methylation profiling may further refine classification [6,29].

### 4.4. Mapping TF/Hormone Patterns to WHO 5th PitNET Subtypes

The WHO 5th edition PitNETs classification incorporates the expression of TFs, hormonal profiles, and clinical status to define specific tumor subtypes. This framework offers a robust basis for diagnosis, prognosis, and treatment decisions. Table 3 provides an overview of the main characteristics of the major PitNET subtypes.

### 4.5. Distinguishing PitNETs from TTF-1–Positive Posterior Pituitary Tumors

There is a critical diagnostic distinction between PitNETs and PPTs. Pituicytes give rise to non-neuroendocrine neoplasms, or PPTs, that include pituicytoma, spindle cell oncocytoma (SCO), granular cell tumor (GCT), and ependymal pituicytoma. These tumors share strong nuclear expression of Thyroid Transcription Factor 1 (TTF-1), which is highly characteristic of posterior pituitary (pituicyte) tumors in the sellar region [14,28].

PPTs consistently express nuclear TTF-1 and are negative for pituitary hormones and lineage-defining TFs (PIT1, TPIT, SF-1), in contrast to PitNETs. Neuroendocrine markers such as synaptophysin and chromogranin A may be weak or variable, reflecting their pituicyte/glial-like nature rather than true adenohypophyseal origin [14,28,30]. On the other hand, PitNETs are almost invariably TTF-1 negative. Demonstration of TTF-1 positivity is especially important in spindle cell or oncocytic sellar tumors, in which SCO could otherwise be mistaken for an oncocytic null cell adenoma or thyrotroph PitNET [14,28]. Recognizing PPTs as TTF-1–positive, non-adenohypophyseal tumors is crucial for accurate classification and prognostication, and prevents mislabeling them as PitNETs.

## 5. Molecular and Clinicopathologic Correlates

In this section, we summarize how recurrent germline and somatic alterations, proliferation indices, and lineage-associated histotypes map onto TF-defined PitNET lineages, and how these molecular patterns relate to imaging features, clinical behavior, and treatment response.

### 5.1. Genetic and Epigenetic Features by Lineage

PIT1-lineage PitNETs consist of somatotroph, lactotroph, and thyrotroph tumors with different but closely related molecular signature characteristics [22]. The PIT1-lineage has a distinctive pattern of diffuse DNA hypomethylation and chromosomal instability in comparison to other groups [31]. Approximately 30–40 percent of somatotroph PitNETs have heterozygous gain-of-function mutations in the *GNAS* gene that increase the Cyclic Adenosine Monophosphate (cAMP) production, leading to an increase in cell proliferation activity [20,32]. Some studies suggest that *GNAS*-mutated somatotroph PitNETs may show higher expression of DRD2 and somatostatin receptors, which could influence responsiveness to medical therapy [20]. Most lactotroph PitNETs are sporadic. A subset of prolactinomas harbors somatic variants, including mutations in the splicing factor gene SF3B1 (Splicing Factor 3B Subunit 1), which have been associated with more aggressive clinical behavior and reduced responsiveness to standard treatment. These tumors also express higher levels of DRD2. Thyrotroph PitNETs have increased Wnt4 gene expression and subsets of the Somatostatin Receptor (SSTR) [20]. On the other hand, germline mutations in the Aryl hydrocarbon receptor Interacting Protein (*AIP*) gene in the PIT1-lineage have been linked to the aggressive and large, sparsely granulated somatotroph tumors in the young patients who in most cases are resistant to standard medical treatment [20,23,32].

TPIT-lineage PitNETs have recurring somatic mutations within the *USP8* gene found in around 20–60 percent of corticotrophic tumors [19,20,22]. These gain-of-function mutations avoid the breakdown of epidermal growth factor receptor (EGFR), which increases the transcription of POMC and leads to ACTH hyper-secretion [19,20,22,32]. In the *USP8* wild-type, regular mutations of USP48 and BRAF (V600E) have been observed, which also increase POMC promoter activity [19,22,32]. In the case of aggressive or metastatic TPIT-lineage tumors, mutations in ATRX (Alpha Thalassemia/Mental Retardation Syndrome X-Linked) and TP53 (Tumor Protein p53) are becoming important indicators of poor clinical behavior [6].

To date, SF-1-lineage PitNETs have not been linked to a single dominant recurrent driver mutation; their somatic mutational landscape appears more heterogeneous [22]. Nevertheless, these tumors do not share methylation clusters with other PitNETs and are often hypermethylated to silence tumor suppressor genes such as *MEG3* (Maternally Expressed Gene 3) [20,22]. However, some somatotroph PitNETs and even corticotroph PitNETs can have gonadotroph signatures [31]. Although it is usually indolent, fast-growing gonadotroph tumors exhibit selective mRNA expression of genes associated with epithelial-mesenchymal transition (EMT) [15,22].

### 5.2. Proliferation, High-Risk Histotypes, and Aggressiveness

The WHO has recognized that certain histopathological subtypes of PitNETs are associated with a higher likelihood of invasion, incomplete resection, and postoperative recurrence, many of which fall within the PIT1- and TPIT-lineages [6,20,27]. In the PIT1-lineage, sparsely granulated somatotroph PitNETs are considered clinically unfavorable variants: they typically present as larger macroadenomas on imaging, show more frequent cavernous sinus invasion, and have lower rates of surgical remission and weaker responses to first-generation somatostatin analogs compared with densely granulated somatotroph tumors [27]. In the TPIT-lineage, silent corticotroph tumors are clinically aggressive, usually presenting as non-functioning macroadenomas with a high rate of cavernous sinus invasion at MRI and a substantial risk of postoperative recurrence, especially when compared with other clinically non-functioning PitNETs such as silent gonadotroph tumors [15,19,20]. Crooke cell tumor is a rare but particularly aggressive type of TPIT-lineage tumor, which is marked by extensive Crooke hyaline change and shows high invasive growth and therapeutic resistance [5,19,20,27]. High-risk histotypes also include plurihormonal PIT1-lineage tumors, particularly the immature PIT1-lineage tumor (previously referred to as silent subtype 3), which are often very large, radiologically invasive lesions that recur frequently despite apparently adequate resection, especially in younger patients [5,12,20]. Typical MRI appearances and signal characteristics of the major lineage-defined histotypes are summarized in Table 4.

Aggressive behavior in these histotypes is frequently associated with elevated proliferative indices (e.g., Ki-67 ≥ 3% and increased mitotic activity), and higher proliferation often parallels more invasive growth and earlier recurrence [15]. However, clinically aggressive behavior can also be observed in tumors with relatively low proliferation, underscoring that clinicoradiologic features, proliferative markers, and lineage-defined histotype must be interpreted together when assessing prognosis [15].

### 5.3. Treatment Implications

Determination of the lineage forms a critical framework for classifying therapeutic interventions for PitNETs. PIT1-lineage tumors express different subtypes of SSTR. Densely granulated somatotroph tumors typically have high SSTR2 expression and usually show the best response to first-generation somatostatin analogs (SSAs), such as octreotide and lanreotide [10,22,32]. In contrast, sparsely granulated somatotroph tumors have less SSTR2 expression and are therefore less sensitive to first-generation SSAs. In these circumstances, they have a better response to second-generation analogs like pasireotide or GH receptor antagonists like pegvisomant [22,32]. Lactotroph PitNETs are uniquely characterized by strong expression of ER*α* in the cell, which is associated with high DRD2 expression, which explains their high sensitivity to dopamine receptor agonists like cabergoline [22,33].

In the TPIT-lineage, corticotroph tumors express SSTR5 and relatively low levels of SSTR2, as chronic hypercortisolism downregulates SSTR2 expression [32,34]. This receptor expression justifies the existing state of pasireotide being the only pituitary-targeting therapeutic agent approved for Cushing’s disease [22,34]. EGFR-targeted therapy, such as gefitinib in tumors with *USP8* mutations, has shown potential in attenuating ACTH secretion and cell proliferation in preclinical studies [19,22,32].

In all the lineages, temozolomide remains the first-line systemic therapy for aggressive and metastatic PitNETs, and therapy outcome has a reverse relationship with a profile of O6-methylguanine DNA methyltransferase (MGMT) expression [10,27,33]. The main lineage-defined histotypes with unfavorable clinical behavior, together with their characteristic molecular alterations and therapeutic implications, are summarized and compared in Table 5.

## 6. Diagnostic Challenges and Pitfalls

Despite the robustness of TF-based classification, several recurrent diagnostic challenges remain; this section discusses triple-negative cases, multilineage or mixed-TF tumors, discordant hormone–TF profiles, and the distinction between null cell PitNETs and non-PitNET sellar lesions, providing practical strategies to avoid misclassification.

### 6.1. TF-Negative or “Triple Negative” Neoplasms

TF-based classification has significantly reduced tumor types that were previously referred to as null-cell adenomas [5]. In the current 5th WHO era, true null-cell PitNETs, characterized by the total absence of PIT1, TPIT, and SF1, are very rare, approximately one percent or less of pituitary neoplasms [35]. Most of the tumors that were once classified as null-cell adenomas have been reclassified as silent gonadotroph PitNETs with the help of SF1 IHC [10]. When a tumor has a triple-negative phenotype, technical factors should be ruled out first because poor fixation or failure to retrieve antigens may result in false-negative nuclear staining [9]. After technical variables are excluded, non-adenohypophyseal sellar lesions that mimic PitNET architecture, such as PPTs and metastatic NENs, should be included in the differential diagnosis [5,17]. Rarely, true TF negative PitNETs can be highly undifferentiated or primitive neoplasms with no markers of terminal differentiation [9]. In these situations, re-staining using optimized protocols and an extended IHC panel (that includes GATA3, NeuroD1) can clarify a diagnosis [12]. In ambiguous triple-negative cases, DNA methylation profiling has demonstrated a powerful supplementary method, being able to put the tumors under discrete classes of molecules even in the absence of lineage-specific protein expression [6,29].

### 6.2. Multilineage/Mixed-TF Tumors

One major area of diagnostic challenge in the 5th WHO era is PitNETs that express more than a single lineage-defining TF in a monomorphic population of tumor cells. This is sometimes referred to as lineage infidelity. Among multilineage PitNETs, co-expression of PIT1 and SF-1 is one of the patterns most frequently described, particularly within PIT1-lineage tumors [29]. While some cases may have double adenomas or collision lesions where two different tumors co-exist, other cases show diffuse co-expression in every tumor cell [12]. These unusual tumors are usually classified within the spectrum of mixed or plurihormonal PitNETs, although their optimal taxonomic position is still under discussion [5].

Most recent epigenetic results, especially relating to DNA methylation profiling, suggest that PIT1/SF1 co-expressing tumors (recently proposed as somatogonadotroph PitNETs) are molecular clusters that are dissimilar to the conventional somatotroph PitNETs [26,29,31]. Clinically, these multi-lineage PitNETs, particularly those that co-express PIT1/SF1, are linked to the more aggressive phenotype, such as higher cavernous sinus invasion rates (45–82 percent) and a higher probability of postoperative recurrence [29]. Nevertheless, the existing data is still scarce, and more longitudinal studies are needed to give a clear picture of the real prognostic value of multilineage PitNETs.

### 6.3. Discordant Hormone and TF Profiles

Discordance between hormone IHC and TF expression is a common diagnostic situation that requires a careful combination of morphologic, immunophenotypic, and clinical evidence. Common discordant patterns include hormone-negative tumors that express lineage-defining TFs [15]. In such cases, TF expression indicates transcriptional lineage commitment, and the absence or low levels of hormone staining may be caused by an inefficient production of hormones, a high rate of intracellular degradation, or a failure to form secretory granules [1,5]. On the other hand, tumors that are hormone positive, yet lack the anticipated TF, are more likely to be due to technical issues, including, but not limited to, poor fixation, low sensitivity of antibodies, or non-specific staining of entrapped non-neoplastic adenohypophyseal cells [9]. The 5th WHO classification establishes a hierarchical method of diagnosis in which TF expression is more important than hormone immunoreactivity in assigning the lineage [1,5]. The diagnosis of diffuse nuclear TPIT expression is therefore adequate to make a diagnosis of silent corticotroph PitNETs in the absence of ACTH immunoreactivity or with a focal localization of ACTH immunoreactivity. However, the results of hormones and TFs should be considered within the broader clinical and biochemical context to differentiate the truly silent tumors from lesions related to subtle or intermittent hormonal hypersecretion.

### 6.4. Differentiating Lineage-Unassigned PitNETs from Non-PitNET Sellar Tumors

Diagnosis of a true PitNET as opposed to non-adenohypophyseal sellar neoplasms is a difficult task. Since the definition of null-cell PitNETs is based on exclusion, histopathologists have to carefully remove mimickers, such as pituicytoma, spindle -cell oncocytoma, and granular-cell tumor [5,17,28]. These posterior pituitary neoplasms consistently show nuclear TTF-1 expression and lack pituitary hormone and PitNET TF expression; neuroendocrine markers such as synaptophysin may be weak or variable. TTF-1 is, therefore, a highly useful discriminatory marker in the differential diagnosis of PitNETs. A broad IHC panel is required in the comprehensive diagnostic workup of a TF/hormone-negative sellar mass. TTF-1 is critical for identifying posterior pituitary lineage, GFAP for glial or pituicyte-derived lesions, and EMA (Epithelial Membrane Antigen) for ependymal pituicytomas or meningiomas [14,28]. Metastatic NENs should also be systematically ruled out, as they tend to express synaptophysin and chromogranin A like PitNETs but lack pituitary-specific TFs. But they express site-specific markers like CDX2 (Caudal Type Homeobox 2) in the intestine and TTF1/Napsin A in the lung [36,37]. An additional diagnostic pitfall arises when a non-pituitary neoplasm entraps normal adenohypophyseal tissue, and the tissue may appear as multilineage differentiation. As a result, it becomes important to assess the reticulin or collagen IV framework, which is usually disrupted by PitNETs, but is preserved by non-neoplastic pituitary tissue entrapped within the acinar space [5]. Combination of morphology, IHC, and structural evaluations is crucial to avoid misclassification and provide correct diagnoses in these cases, which are difficult to diagnose.

## 7. Implementation in Reporting and Future Directions

This section provides pragmatic guidance on incorporating TF-based lineage classification into routine pathology reporting, outlines a minimum dataset and suggested diagnostic wording, and discusses how these practices interface with multidisciplinary management and emerging tools such as methylation profiling, digital pathology, and artificial intelligence.

### 7.1. How to Report: Minimum Dataset and Suggested Wording

The 5th edition of the WHO Classification of Endocrine and Neuroendocrine Tumors emphasized that pituitary pathology reports should integrate morphologic, immunophenotypic, and relevant clinical information. At least, pituitary tumor reports should include the description of morphological characteristics of the adenohypophyseal cell population, as well as the findings of a comprehensive IHC panel [5,9]. In practical terms, a minimum dataset for reporting PitNETs should include the following elements:

- Basic clinical context (where available): patient age and sex, functional status (clinically functioning vs. non-functioning), and main presenting syndrome.
- Tumor site and extent: sellar/suprasellar location, approximate size, and, where assessable, evidence of invasion into adjacent structures (e.g., cavernous sinus, sphenoid).
- Morphology of the adenohypophyseal component: growth pattern (diffuse, papillary, trabecular, etc.), cytologic features (acidophilic, basophilic, chromophobic), granulation pattern (densely vs. sparsely granulated where applicable), and characteristic features such as fibrous bodies or Crooke hyaline change.
- Immunophenotype—core neuroendocrine and lineage markers:
  o Pan-neuroendocrine markers (e.g., synaptophysin, chromogranin A).
  o Lineage-defining transcription factors (PIT1, TPIT, and SF1), with documentation of their nuclear staining pattern and intensity.
  o Auxiliary markers when used (e.g., ERα and GATA3) to support lineage assignment in difficult cases.
- Immunophenotype—pituitary hormones and adjunct markers:
  o The six anterior pituitary hormones (GH, PRL, ACTH, TSH, FSH, and LH) and the α-subunit, indicating which are positive and whether staining is diffuse vs. focal [9].
  o LMWCK, particularly CAM5.2, to detect perinuclear fibrous bodies in sparsely granulated somatotroph PitNETs and other subtypes with distinctive cytoskeletal features [5,27].
- Proliferation assessment:
  o Ki-67 labelling index, measured in hotspots and reported as a percentage (or number of positive cells per mm2) [26].
  o Mitotic activity, if counted, is expressed as mitoses per 10 high-power fields.
- Additional markers:
  o p53 immunostaining, recognizing that its independent prognostic value is debated but that diffuse strong staining may suggest underlying genomic instability in clinically aggressive tumors [12,27].

The final diagnostic line should follow the WHO 5th edition terminology and designate the lesion as a PitNET with a specific histological subtype (e.g., ‘immature PIT1-lineage PitNET’). A diagnosis of null cell adenoma should be reserved as a diagnosis of exclusion for tumors that are negative for all lineage-defining TFs and pituitary hormones, after confirming adequate technical quality and excluding posterior pituitary tumors and other non-adenohypophyseal sellar lesions [5].

### 7.2. Impact on Multidisciplinary Management

The correct TF-based lineage and subtype assignment have a direct implication on the postoperative management and long-term monitoring, which enforces the cooperation between pathologists, endocrinologists, neurosurgeons, and oncologists [26,27]. In the PIT1 lineage, subtyping can be refined, especially in relation to making therapeutic decisions. Densely granulated somatotroph PitNET, as indicated by a strong expression of SSTR2, responds strongly to first-generation SSAs, but sparsely granulated ones with lower levels of SSTR2 and relative predominance of SSTR5 may need second-generation agents, like pasireotide or GH receptor antagonists like pegvisomant [5,27]. Similarly, in lactotroph PitNET, ERα expression correlates with high DRD2 and reliably predicts sensitivity for dopamine agonists [33]. The lineage assignment also helps to classify high-risk subtypes such as silent corticotroph PitNETs, Crooke cell tumors, and immature PIT1-lineage PitNETs. They have a notably high proportion of cavernous sinus invasion, early recurrence, and diminished response to standard medical treatment. These patients often warrant closer postoperative monitoring with more frequent Magnetic Resonance Imaging (MRI) follow-up and earlier consideration of adjuvant radiotherapy or systemic treatment, such as temozolomide [35,38].

Moreover, certain IHC and morphological patterns suggest the review of underlying genetic syndromes. The presence of sparsely granulated somatotroph PitNETs in young patients should make physicians consider *AIP* mutations, whereas multifocal PIT1-lineage tumors might indicate MEN1 syndrome [5,26]. Thus, accurate pathological diagnosis does not only inform immediate management, but also informs genetic counseling, management follow-up strategies, and clinical trial eligibility.

### 7.3. Future Perspectives

The future of pituitary pathology lies in the adoption of TF-based histopathology combined with pangenomic methods, particularly, DNA methylation [20]. Methylation profiling has emerged as a molecular gold standard and can solve the diagnostically challenging cases, such as TF-negative, multilineage, or poorly differentiated PitNETs whose diagnosis is uncertain with conventional IHC [12,29].

Routine diagnosis is expected to further transform along with advancement in digital pathology and artificial intelligence. TF expression, Ki-67 labeling indices, and mitotic activity can be objectively measured with automated image analysis systems and decrease the extensive inter-observer variability introduced with manual scoring [1,26]. Furthermore, the recent machine-learning models that combine histopathology with radiologic characteristics have shown a promise and capability in predicting specific subtypes such as densely versus sparsely granulated somatotroph PitNETs, and even TF-defined lineage patterns pre-operatively, which may inform the surgical planning and initial therapeutic choices [26].

Despite such advances, the extensive clinical application is dependent on standardization. TF antibody clone harmonization, consensus IHC positivity thresholds, and robust inter-laboratory quality control initiatives will be necessary to provide diagnostic reproducibility [13,26]. With such technical and logistical obstacles resolved, a combined morphologic, immunophenotypic, and molecular hierarchy will redesign precision diagnostics and customized therapy.

## 8. Conclusions

The conversion to a TF-based classification system has become the essential foundation of modern diagnostic PitNET. Routine assessment of the core TFs (PIT1, TPIT, and SF1), together with auxiliary markers such as GATA3 and ERα, has allowed pathologists to move away from purely subjective morphologic criteria. This approach has markedly reduced the size of the ambiguous null cell adenoma, with many cases now reclassified as silent gonadotroph PitNETs or other lineage-specific neoplasms. Lineage-level subtyping can be used to recognize high-risk variants early, whose unique biological features demand heightened surveillance and multidisciplinary interventions. Despite these advances, there are still diagnostic challenges, including handling of highly rare cases of so-called triple-negative neoplasms and tumors with multilineage infidelity, which today push the limits of classical classifications, and potentially have a more aggressive clinical behavior. Future developments will require integration of pangenomic signatures—such as DNA methylation and transcriptomic profiling—with digital pathology and AI-based image analysis to standardize IHC interpretation and reduce inter-observer variability. As these tools are validated and increasingly adopted, refined lineage-based and molecular characterization of PitNETs is expected to move clinical practice away from one-size-fits-all approaches. This should support more accurate prognostication and progressively more personalized management of patients with PitNETs.

## Figures and Tables

**Table 1:** Evolution of WHO classification of pituitary tumors [16].

Category	2004 WHO	2017 WHO	2022 WHO (5th ed.)
Terminology	Adenoma	Adenoma vs. tumor vs. PitNET	Pituitary Neuroendocrine Tumor (PitNET)
Ihc Basis	Hormonal	Transcription factors (TF) and hormonal	Transcription factors (TF) & hormonal
Type/Lineage	‘Typical’	SF1 lineage Gonadotroph	SF1 lineage Gonadotroph
	‘Atypical’	TPIT lineage Corticotroph PIT1 lineage Lactotroph (sparsely granulated, densely granulated, ASC) Somatotroph (sparsely granulated, densely granulated, mammosomatotroph, mixed somatotroph–lactotroph) Thyrotroph Plurihormonal (PIT1-positive plurihormonal ^a^, unusual combinations)	TPIT lineage Corticotroph PIT1 lineage Lactotroph (sparsely granulated, densely granulated) Somatotroph (sparsely granulated, densely granulated) Mammosomatotroph ^b^ Mixed somatotroph and lactotroph ^b^ Thyrotroph Mature plurihormonal PIT1 lineage ^c^ Immature PIT1 lineage ^c^ Acidophil stem cell ^b^
No Distinct Cell Lineage	Null cell	Null cell	Null cell Plurihormonal
Proliferative Markers	Ki-67 > 3% Elevated mitotic index p53 ↑	Ki-67 > 3% Elevated mitotic index —	— — —
Carcinoma/Metastasis	Craniospinal or distant metastases	Craniospinal or distant metastases	Term omitted; replaced with “Metastatic PitNET” ^c^

^a^—Newly defined in 2017 WHO classification; ^b^—Newly described as separate ‘type’ rather than ‘subtype’ in 2022 WHO classification; ^c^—Newly defined in 2022 WHO classification. ASC, acidophil stem cell; IHC, immunohistochemistry; TF, transcription factor; ↑ increased expression.

**Table 2:** Recommended minimal and extended TF-IHC panel.

Markers	Primary Function
**Minimal Panel**	
PIT1	Lineage marker for somatotroph, lactotroph, and thyrotroph cells
TPIT	Lineage marker for corticotroph cells
SF1	Lineage marker for gonadotroph cells
Synaptophysin	Pan-neuroendocrine marker to confirm neuroendocrine differentiation; highly sensitive
Chromogranin A	Pan-neuroendocrine marker; less sensitive than synaptophysin
Ki-67 (MIB1)	Proliferation marker to assess tumor growth potential
**Extended Panel**	
GATA3	Marker for thyrotroph and gonadotroph
ERα	Marker for lactotroph and mammosomatotroph
CAM5.2	Distinguishes high-risk subtypes; detects fibrous bodies of sparsely granulated somatotroph PitNETs

**Table 3:** TF-based classification of PitNETs according to WHO 5th edition. Adapted from WHO and recent studies [5,14,25].

PitNET Subtype	Transcription Factor(s)	Hormone IHC Pattern	Clinical/Functional Status	Key Comments
Densely Granulated Somatotroph PitNET	PIT1+	GH+, α-subunit+	Acromegaly	Acidophilic cells; diffuse GH staining; perinuclear LMWCK; usually responsive to SSA
Sparsely Granulated Somatotroph PitNET	PIT1+	GH weak/focal	Acromegaly or clinically NF	High-risk subtype; chromophobic cells; fibrous bodies (CAM5.2+); aggressive behavior and reduced SSA response
Lactotroph PitNET	PIT1+, ERα+	PRL+	Hyperprolactinemia	Most are sparsely granulated with juxtanuclear “dot-like” PRL; excellent dopamine agonist response
Thyrotroph PitNET	PIT1+, GATA3+	TSH+, α-subunit+	Hyperthyroidism (rare)	Often fibrotic or spindle-celled; rare but diagnostically challenging
Corticotroph PitNET	TPIT+	ACTH+	Cushing’s disease	Basophilic cells; strong PAS positivity; typically, microadenomas
Crooke Cell Tumor	TPIT+	ACTH+ (often weak)	Cushing’s disease or NF	High-risk variant; perinuclear hyaline keratin rings; aggressive and invasive
Silent Corticotroph PitNET	TPIT+	ACTH+	Clinically NF	High-risk; larger size, invasive growth, higher recurrence rate than gonadotroph tumors
Gonadotroph PitNET	SF1+, GATA3+ (±ERα)	FSH/LH/α-subunit (often weak or negative)	Clinically NF	Most common PitNET subtype; diffuse nuclear SF-1 is the most reliable marker
Mature Plurihormonal PIT1-Lineage PitNET	PIT1+	Multiple PIT1-lineage hormones	Variable (often functional)	Well-differentiated cells; distinct from aggressive immature subtype
Immature PIT1-Lineage PitNET	PIT1+ (ERα/GATA3 variable)	Multihormonal (focal/patchy)	Clinically NF or mixed	High-risk; lineage infidelity; large, invasive tumors; formerly “silent subtype 3”
Acidophil Stem Cell PitNET	PIT1+	Usually GH+, PRL+	Acromegaly, Hyperprolactinemia	High-risk; scattered fibrous bodies
Null Cell PitNET	PIT1−/TPIT−/SF-1−	No hormone expression	Clinically NF	Diagnosis of exclusion; very rare with modern TF IHC
Mixed/plurihormonal PitNET	Multiple combination	Multiple combination	Variable	Variable

**Table 4:** Typical MRI features of major TF-defined PitNET lineages and clinically relevant histotypes.

Lineage/Histotype	Typical Clinical Context	Usual Tumor Size at MRI	MRI Signal and Enhancement Pattern *	Invasion/Recurrence Pattern ^†^
**PIT1-lineage—densely granulated somatotroph**	Acromegaly with biochemically active GH excess	Often microadenoma or small macroadenoma	Usually iso- to mildly hypointense on T1 and iso- to mildly hyperintense on T2; relatively homogeneous, strong contrast enhancement	Less frequent cavernous sinus invasion; higher surgical remission rates compared with sparsely granulated somatotroph PitNETs [27]
**PIT1-lineage—sparsely granulated somatotroph**	Acromegaly; often younger patients; treatment-resistant disease	Typically larger macroadenoma	Macroadenoma, often heterogeneous on T2 (cystic or hyperintense foci); robust enhancement but more irregular due to size and internal heterogeneity	More frequent cavernous sinus invasion and lower rates of gross total resection and biochemical remission than densely granulated tumors [27]
**PIT1-lineage—lactotroph**	Hyperprolactinemia (amenorrhea, galactorrhea, hypogonadism)	Microadenoma or macroadenoma	Signal characteristics are usually similar to other PitNETs (iso- to hypointense on T1, iso- to hyperintense on T2) with homogeneous or mildly heterogeneous enhancement	Invasive macroprolactinomas may extend into the cavernous sinus, but many lesions are resectable or controlled medically [5,20]
**PIT1-lineage—immature PIT1-lineage/plurihormonal**	Often clinically non-functioning or mixed hormone profiles; younger patients	Large macroadenoma or giant adenoma	Bulky, lobulated masses with heterogeneous T2 signal and enhancement, reflecting cystic change, hemorrhage, or necrosis	Frequently radiologically invasive with cavernous sinus and suprasellar extension and high postoperative recurrence rates [5,12,20]
**TPIT-lineage—functioning corticotroph (Cushing’s disease)**	Overt hypercortisolism; ACTH-dependent Cushing’s disease	Often microadenoma; occasionally small macroadenoma	Small, often subtle lesions; iso- or hypointense on T1, variably hyperintense on T2; may enhance less than normal gland on dynamic sequences	Limited local invasion in many cases, but residual/recurrent disease can occur, especially when the lesion is not well visualized preoperatively [14,19]
**TPIT-lineage—silent corticotroph**	Clinically non-functioning macroadenoma	Usually macroadenoma	Large, solid sellar/suprasellar mass with fairly typical PitNET signal (iso- to hypointense T1, iso- to hyperintense T2) and strong, sometimes heterogeneous enhancement	High rate of cavernous sinus invasion and postoperative recurrence compared with other clinically non-functioning PitNETs, especially SF1-lineage tumors [15,19,20]
**TPIT-lineage—Crooke cell PitNET**	Often aggressive corticotroph tumor; may present with Cushing’s disease or as non-functioning mass	Macroadenoma, frequently large	Macroadenoma with variable signal intensity and heterogeneous enhancement; no pathognomonic signal feature but often invasive at presentation	Marked local invasiveness and therapeutic resistance; high recurrence rates even after apparently adequate resection [5,19,20,27]
**SF1-lineage—gonadotroph PitNET**	Clinically non-functioning macroadenoma; symptoms from mass effect	Typically macroadenoma	Conventional PitNET appearance: iso- to hypointense on T1, iso- to moderately hyperintense on T2, with homogeneous or mildly heterogeneous enhancement	Often sizeable at diagnosis but with more indolent clinical courses and lower recurrence rates than silent corticotroph or immature PIT1-lineage PitNETs [14,20,22]

* MRI signal descriptions are general patterns; individual lesions may show variability due to cystic change, hemorrhage, or prior treatment. ^†^ Invasion and recurrence patterns are summarized from clinicopathologic series focusing on TF-defined histotypes [5,12,14,15,19,20,22,27]; specific imaging–outcome correlations should be interpreted in the context of individual studies.

**Table 5:** Key molecular drivers, lineage-defined histotypes with unfavorable clinical behavior, and main therapeutic implications in TF-based PitNET classification. Adapted from WHO classification and recent studies [17,22,23].

Tf-Defined Group	Typical Molecular Drivers/Alterations	High-Risk Histotypes (WHO 5th)	General Therapeutic Considerations
**PIT1 Lineage**	Somatic (*GNAS*, SF3B1, PRLR); Germline (*AIP*, MEN1)	Sparsely granulated somatotroph; Immature PIT1-lineage PitNET; Acidophil stem cell PitNET	SSR–directed therapy (octreotide, pasireotide); GH receptor antagonists (pegvisomant) in resistant acromegaly; dopamine agonists for lactotroph tumors
**TPit Lineage**	*USP8*, USP48, BRAF, ATRX; additional signaling pathway alterations reported	Silent corticotroph PitNET; Crooke cell tumor	Pasireotide preferred due to SSTR5 expression; emerging molecularly targeted approaches are under investigation in selected cases
**SF-1 Lineage**	No recurrent somatic driver mutations identified; epigenetic alterations (*MEG3*)	Rare aggressive gonadotroph PitNETs; most clinically non-functioning	Surgery remains the primary therapy; with limited and variable responses to medical therapies
**TF-Negative/Mixed PITNets**	Copy number alterations; TP53 abnormalities reported in aggressive disease	Metastatic PitNET	Temozolomide as first-line systemic therapy; treatment response associated with low MGMT expression

## Data Availability

No new data were created or analyzed in this study. Data sharing is not applicable to this article.

## Abbreviations

The following abbreviations are used in this manuscript:

ACTH	Adrenocorticotropic Hormone
AIP	Aryl Hydrocarbon Receptor–Interacting Protein
ASC	Acidophil Stem Cell
ATRX	Alpha Thalassemia/Mental Retardation Syndrome X-Linked
BRAF	B-Raf Proto-Oncogene
cAMP	Cyclic Adenosine Monophosphate
CDX2	Caudal Type Homeobox 2
DRD2	Dopamine Receptor D2
EGFR	Epidermal Growth Factor Receptor
EMA	Epithelial Membrane Antigen
EMT	Epithelial–Mesenchymal Transition
ERα	Estrogen Receptor Alpha
FSH	Follicle-Stimulating Hormone
GATA3	GATA Binding Protein 3
GCT	Granular Cell Tumor
GFAP	Glial Fibrillary Acidic Protein
GH	Growth Hormone
GNAS	Guanine Nucleotide-binding Protein alpha stimulating
H&E	Hematoxylin and Eosin
IHC	Immunohistochemistry
LH	Luteinizing Hormone
LMWCK	Low Molecular Weight Cytokeratin
MAPK	Mitogen-Activated Protein Kinase
MEG3	Maternally Expressed Gene 3
MEN1	Multiple Endocrine Neoplasia Type 1
MGMT	O6-Methylguanine-DNA Methyltransferase
MRI	Magnetic Resonance Imaging
NEN	Neuroendocrine Neoplasm
NF	Non-Functioning
NF-PitNET	Non-Functioning Pituitary Neuroendocrine Tumor
NR5A1	Nuclear Receptor Subfamily 5 Group A Member 1
PA	Pituitary Adenoma
PAS	Periodic Acid–Schiff
PIT1	Pituitary-Specific Positive Transcription Factor 1
POU1F1	POU Class 1 Homeobox 1
PitNET	Pituitary Neuroendocrine Tumor
PITX1	Pituitary Homeobox 1
PITX2	Pituitary Homeobox 2
POMC	Pro-opiomelanocortin
PROP1	Prophet of PIT1
PRL	Prolactin
PRLR	Prolactin Receptor
PPT	Posterior Pituitary Tumor
SCT	Silent Corticotroph Tumor
SCO	Spindle Cell Oncocytoma
SF1	Steroidogenic Factor 1
SF3B1	Splicing Factor 3B Subunit 1
SSTR	Somatostatin Receptor
SSTR2	Somatostatin Receptor Type 2
SSTR5	Somatostatin Receptor Type 5
SSA	Somatostatin Analogue
TBX19	T-Box Transcription Factor 19
TF	Transcription Factor
TP53	Tumor Protein p53
TPIT	T-Box Pituitary Transcription Factor
TSH	Thyroid-Stimulating Hormone
TTF-1	Thyroid Transcription Factor 1
USP8	Ubiquitin-Specific Protease 8
USP48	Ubiquitin-Specific Protease 48
V600E	Valine-to-Glutamic Acid Substitution at Codon 600
WHO	World Health Organization
